# *Munroa argentina*, a Grass of the South American Transition Zone, Survived the Andean Uplift, Aridification and Glaciations of the Quaternary

**DOI:** 10.1371/journal.pone.0128559

**Published:** 2015-06-25

**Authors:** Leonardo D. Amarilla, Ana M. Anton, Jorge O. Chiapella, María M. Manifesto, Diego F. Angulo, Victoria Sosa

**Affiliations:** 1 Instituto Multidisciplinario de Biología Vegetal (IMBIV), Consejo Nacional de Investigaciones Científicas y Técnicas (CONICET)–Universidad Nacional de Córdoba, Vélez Sarsfield 1611–X5016GCA, 5000 Córdoba, Argentina; 2 Instituto de Recursos Biológicos, INTA, Castelar, 1712 Prov. de Buenos Aires, Argentina; 3 Biología Evolutiva, Instituto de Ecología AC, Carretera antigua a Coatepec 351, El Haya, 91070 Xalapa, Veracruz, Mexico; Kunming Institute of Botany, CHINA

## Abstract

The South American Transition Zone (SATZ) is a biogeographic area in which not only orogeny (Andes uplift) and climate events (aridification) since the mid-Miocene, but also Quaternary glaciation cycles had an important impact on the evolutionary history of the local flora. To study this effect, we selected *Munroa argentina*, an annual grass distributed in the biogeographic provinces of Puna, Prepuna and Monte. We collected 152 individuals from 20 localities throughout the species’ range, ran genetic and demographic analyses, and applied ecological niche modeling. Phylogenetic and population genetic analyses based on cpDNA and AFLP data identified three phylogroups that correspond to the previously identified subregions within the SATZ. Molecular dating suggests that *M*. *argentina* has inhabited the SATZ since approximately 3.4 (4.2–1.2) Ma and paleomodels predict suitable climate in these areas during the Interglacial period and the Last Glacial Maximum. We conclude that the current distribution of *M*. *argentina* resulted from the fragmentation of its once continuous range and that climate oscillations promoted ecological differences that favored isolation by creating habitat discontinuity.

## Introduction

The uplift of the Andes in the Neogene had a strong impact on the evolutionary history of South American biota [1–3]. The rise occurred in discrete periods, progressing from south to north and from west to east [1–5]; once formed, this mountain chain became the sole barrier to atmospheric circulation in the Southern Hemisphere [3,5]. There were two major uplift events, one during the middle Miocene (12 Ma) and the other at the beginning of the Pliocene (5 Ma; [6]). Recent phylogeographic studies have shown that the Andean uplift both created a dispersal route for lineages of organisms and also acted as an evolutionary force favoring rapid diversification via allopatric speciation, habitat fragmentation, and ecological displacement into several habitats including highland and montane forests, and arid and semiarid vegetation [7–18]. The rain shadows resulting from the uplift affected climate [19,20] and formed a narrow region with scant precipitation (<300 mm/year) known as the Arid Diagonal [21] or as the South American Transition Zone (SATZ) [22–25]. Based on the distribution patterns of a number of plant and animal groups, this zone has been subdivided into six biogeographic provinces: North Andean Paramo, Puna, Coastal Peruvian Desert, Atacama, Prepuna, and Monte [22,25].

Late Neogene and Quaternary climate oscillations that involved several ice age cycles influenced species and community distribution patterns on Earth, and although aridification occurred mainly during the Pliocene, fluctuations in climate during the Pleistocene also contributed to the expansion of arid and semiarid regions in South America [7,8,26–33]. These environmental changes also had an effect on microevolutionary processes in species by influencing gene flow, causing demographic expansion/contraction, and by creating genetic bottlenecks [34]. The role of climate oscillations in the evolutionary history of organisms, including the survival of species in refugia, changes in population number, size and genetic variation, and tempo and mode of recolonization, has been documented for North American taxa [15,35–40]. However, far less is known for plant species in the arid-temperate regions of the Southern Hemisphere [41]. Only the southern areas of South America and New Zealand are in geographic positions comparable to the glaciated areas in the Northern Hemisphere. Glaciers in the Southern Hemisphere were mostly restricted to mountain ranges, but at low and middle latitudes, lower average temperatures and variable precipitation regimes probably influenced all types of vegetation as well [42–47].

Recent studies on the evolutionary history of animal and plant taxa in the SATZ provinces [7,32,33,42–49] suggest that their evolutionary processes are more strongly associated with the aridification processes that gave rise to the SATZ, and to the final uplifting of the Southern Andes (5 Ma) than with Quaternary glaciation (1 Ma–15 Ka). In fact, these geological events produced remarkable climatic and landscape changes that promoted habitat fragmentation, inter- and intraspecific differentiation, demographic reduction/expansion and genetic bottlenecks in the different groups of organisms inhabiting the SATZ [33,44]. Of the few studies focusing on understanding the effects of the Late Neogene and Quaternary climate oscillations that occurred in plant species of the SATZ [7,15,41,42,44], three have concentrated on perennial South American species of *Hordeum* [15,39,42], and identified a recent divergence in the Late Pleistocene [15,42]. Therefore they focused solely on understanding responses to Quaternary climatic oscillations, their results however did not find a clear pattern either of expansion or contraction of their distribution ranges [15,42].

To better understand the effect of not only the Quaternary climate oscillations but also of the Late Neogene climate oscillations and the uplift of the Andes in grassland taxa, we selected *Munroa argentina* (Poaceae), an annual [50] grass which diverged before than the perennial South American species of *Hordeum* and with a wide distribution in three SATZ provinces (Puna, Prepuna, and Monte) [50,51]. We had previously identified that this species originated in the Late Pliocene-early Pleistocene at 3.5 (4.3–2.4) Ma, closely related to *M*. *andina*, with a common ancestor that inhabited the southern area of the SATZ [52]. Thus *M*. *argentina* is an ideal subject for studying the effects of these climate oscillations and the Andean uplift because it was settled in this biogeographic region before these events occurred. This species inhabits the open areas of plains and mountains from mid- (1000 m a.s.l.) to high elevations (4200 m a.s.l) on alluvial and sandy-stony soils, along seasonally dry creeks and rivers [50–52]. In the present paper, we analyze whether post-glacial climate fluctuations in the SATZ had an effect on the extent of *M*. *argentina*’s distribution and the structure of its genetic variation. Analyzing these two aspects can help reconstructing the evolutionary history of a species and identify putative Pleistocene refugia [15,37,39], recolonized areas [15,39,42,44], fragmented habitats or migration corridors [15,37,39,41,42,44]. Based on the divergence time of this species we hypothesized that its populations expanded in post-glacial times to occupy the marginal part of its range, concurrent with increased aridity during the Holocene, thus leading to the prediction that the greatest genetic diversity would occur in the ancestral populations from the southernmost area of the SATZ.

## Materials and Methods

### Ethics Statement

Material was collected and deposited in CORD the Herbarium of the Universidad Nacional de Cordoba, Argentina. The herbarium is recognized by the "Secretaría de Ambiente y Desarrollo Sustentable de la Nación" for doing that. Dr. Ana M Anton is the curator of Poaceae and participated in the collection of material. Collections were made under three scientific collecting permits granted by the following government agencies in Argentina “Secretaría de Medio Ambiente y Desarrollo Sustentable” in Salta (Res.091, Expte. 119–10233)”, by the “Secretaría de Ambiente y Desarrollo Sustentable, Subsecretaría de Conservación y Áreas Protegidas, Dirección de Conservación y Áreas Protegidas” in San Juan (Res. 056)” and by the “Administración de Parques Nacionales” (Ley 22351, Autorización de Investigación Proyecto 1249).

### Study species

*M*. *argentina* is a predominantly cross-pollinating annual grass with flowers with exserted anthers [50,51]. Plants are small, up to 15 cm tall, trailing on the ground and stoloniferous, with branches forming rosettes and both female and hermaphrodite flowers on the same plant (gynomonoecy). Flowers are grouped in inflorescences included in the leafy fascicles, comprising 1–4 subsessile spikelets, with the rachilla or secondary rachis disarticulating [50,51]. Single florets or portions of inflorescences can be units of dispersion; its coriaceous and geniculate glumes with awns can attach to animal fur or bird plumage. Thus caryopses or propagules can be dispersed by wind (anemochory), water (hydrochory) or by birds and animals (epizoochory) [52]. *M*. *argentina* has been reported in Argentina, Bolivia and Peru [50,51].

### Sampling, DNA extraction, amplification and sequencing

**Fig 1:**
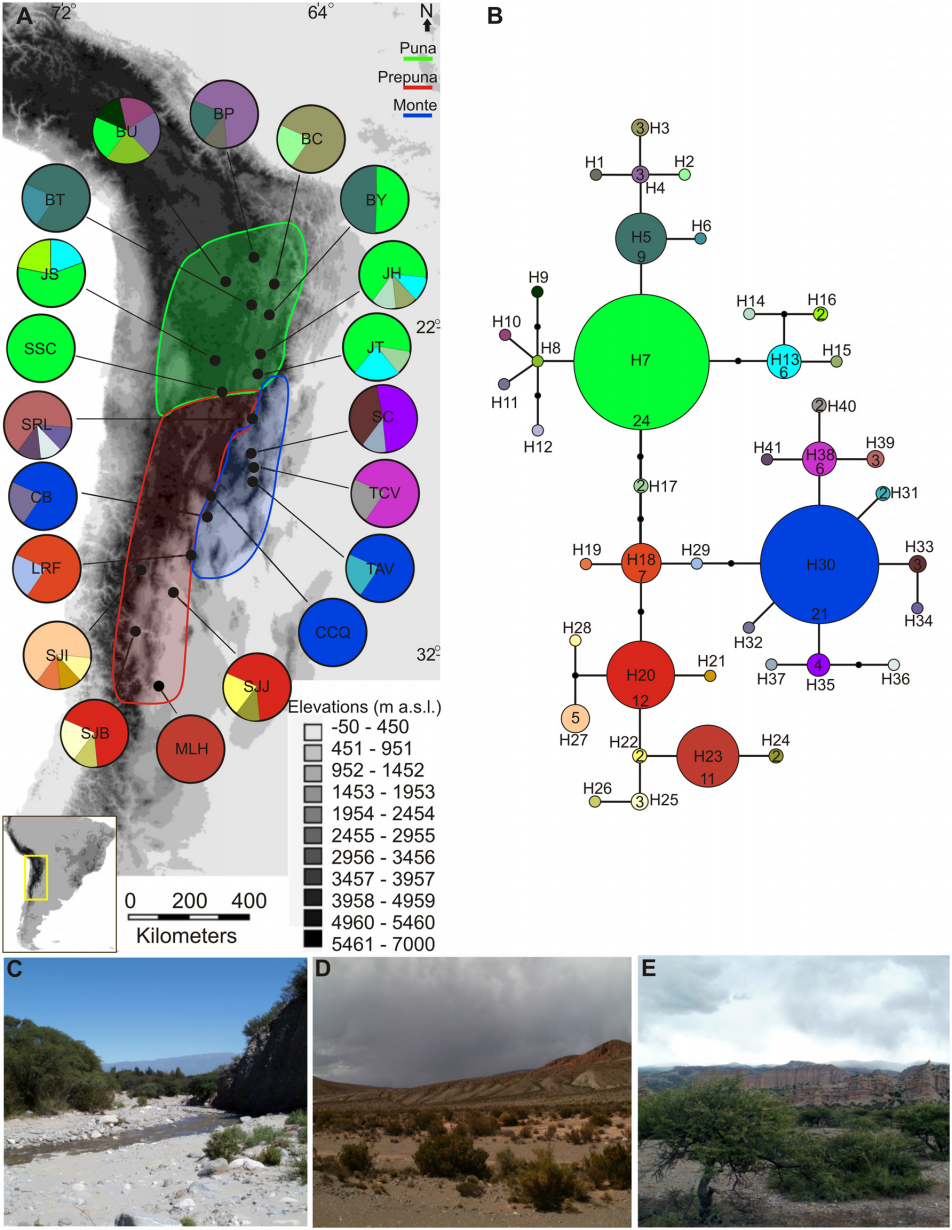
Map of the sampling sites and phylogenetic relationships obtained by analysis of cpDNA haplotypes. **A**, Geographic distribution of *M*. *argentina* cpDNA haplotypes in the South American Transition Zone (SATZ). **B**, Statistical parsimony haplotype network. Three population groups were defined: Puna, Prepuna, and Monte. Pie charts represent the haplotypes found in each sampling locality. Pie chart section size is proportional to the number of individuals per haplotype. Haplotype designations in the network correspond to those given in Table 1 (H1–H41). The numbers in the haplotypes indicate the number of individuals that share that haplotype. **C**, **D** and **E**, habitats where *Munroa* grows: Tucumán (Argentina), San Juan (Argentina), and Potosí (Bolivia), respectively. Photographs by Leonardo Amarilla.

A total of 152 accessions of *M*. *argentina* were sampled from 20 localities that cover its entire distribution range and its elevation gradient from 1000 to 4200 m a.s.l. (Fig 1). Sampling included four to eleven plants per locality, depending on population density. In 16 localities, sampling was carried out in 200 m transects. Vouchers were deposited at CORD, the herbarium of the Universidad Nacional de Córdoba and, when necessary, samples were taken from herbarium specimens (Table 1).

**Table 1:** *M*. *argentina* sampling locations, number of individuals (*N*_ind._), voucher, phenological status of individuals of each locality in February (PS): flowering/fruiting, not flowering (—), biogeographic province, geographic coordinates, and haplotypes detected.

Sample location	Abbreviation	*N*_ind._	Voucher	PS	Province	Latitude/Longitude	Haplotypes
**Argentina, Mendoza, Las Heras**	MLH	11	Amarilla 1B (CORD)	Fruit	Prepuna	-32.7565/-68.8483	H23
**Argentina, San Juan, Bella Vista**	SJB	10	Chiapella 2580 (CORD)	Fruit	Prepuna	-31.1498/-69.4499	H20, 26, 25
**Argentina, San Juan, Jachal**	SJJ	10	Chiapella 2596 (CORD)	Fruit	Prepuna	-30.2998/-68.2773	H20, 24, 22
**Argentina, San Juan, Iglesias**	SJI	8	Peterson 19267 (2 ind.)/ Amarilla 3 (CORD)	Flower/Fruit	Prepuna	-29.5732/-69.4564	H 19, 28, 27, 21
**Argentina, La Rioja, Famatina**	LRF	8	Chiapella 2604 (CORD)	Flower/Fruit	Monte/Prepuna	-28.9155/-67.5164	H 18, 29
**Argentina, Catamarca, Corral Quemado**	CCQ	7	Chiapella 2672 (CORD)	Flower/Fruit	Monte	-27.2281/-66.9374	H 30
**Argentina, Catamarca, Belén**	CB	8	Chiapella 2656 (CORD)	Flower/Fruit	Monte	-27.6498/-67.0330	H 30, 32
**Argentina, Tucumán, Amaicha del Valle**	TAV	9	Amarilla 1A (CORD)	Flower	Monte	-26.5902/-65.9181	H 30, 31
**Argentina, Tucumán, Colalao del Valle**	TCV	8	Amarilla 11 (CORD)	Flower	Monte	-26.3644/-65.9470	H 38, 40
**Argentina, Salta, Cafayate**	SC	8	Chiapella 2712 (CORD)	Flower	Monte	-26.0722/-65.9698	H 33, 35, 37
**Argentina, Salta, Rosario de Lerma**	SRL	6	Chiapella 2697 (CORD)	Flower	Monte	-24.9882/-65.5718	H 34, 36, 39, 41
**Argentina, Salta, S. A. de los Cobres**	SSC	4	Amarilla 31 (CORD)	Flower/—	Prepuna/Puna	-24.2100/-66.3230	H 7
**Argentina, Jujuy, Tilcara**	JT	11	Amarilla 22A (CORD)	Flower	Prepuna/Puna	-23.5788/-65.3963	H 7, 17, 13
**Argentina, Jujuy, Humahuaca**	JH	9	Amarilla 22B (CORD)	Flower	Prepuna/Puna	-23.2097/-65.3512	H 7, 13, 14, 15
**Argentina, Jujuy, Susques**	JS	9	Amarilla 24 (CORD)	Flower/—	Prepuna/Puna	-23.4166/-66.4833	H 7, 13, 16
**Bolivia, Camargo**	BC	4	Wood 9498 (4 ind.)(LPB)	Flower/—	Puna	-20.6416/-65.2093	H 2, 3
**Bolivia, Potosí**	BP	6	López 552(3 ind.)/Asplund 6485 19267 (3 ind.) (LPB)	Flower/—	Puna	-19.6148/-65.7646	H 1, 4, 5
**Bolivia, Uyuni**	BU	5	Chiapella 2801 (CORD)	Flower/—	Puna	-20.4607/-66.8273	H 8, 9, 10, 11, 12
**Bolivia, Tupiza**	BT	5	Chiapella 2818 (CORD)	Flower/—	Puna	-21.4433/-65.7188	H 5, 6
**Bolivia, Yunchara**	BY	6	Beck 26643, 26902/84, (2 ind. per n°) (LPB)	Flower/—	Puna	-21.8236/-65.2364	H 5, 7

Total genomic DNA was isolated from silica-gel-dried leaf tissue using the CTAB method [53]. Primers used in this study were: rpS16-900F & 3914PR for *rps16-trnK*, and ndhAx4 & ndhAx3 for the *ndhA* intron [54]. A single amplification protocol was used to amplify the chloroplast and nuclear regions, following Peterson & al. [54]. Amplification products were visualized under UV light after electrophoretic separation on a 1% agarose TBE gel stained with SYBR Safe gel stain (Invitrogen, Carlsbad, California, U.S.A.). Amplified products were sent to Macrogen Inc. (Seoul, South Korea) for purification and sequencing with the BigDyeTM terminator kit and run on an ABI 3730XL. Sequences were assembled and edited using Sequencher v4.1 (Gene Codes Corporation, Ann Arbor, Michigan, U.S.A.). The sequences were pre-aligned in Mega 5 [55] using Muscle [56] and alignments were subsequently optimized by eye. GenBank accessions are listed in S1 Table.

Amplified fragment length polymorphisms (AFLP) were assayed following the protocol of Lachmuth & al. [57]. After the initial screening of 12 primer combinations, we selected six primer combinations for the final analysis: AAT (FAM)-AAA, ACT (FAM)-AAA, AAT (FAM)-AAC, AAA (FAM)-AAA, AAA (FAM)-AAC and ACT (FAM)-AAC. Fragment analysis was performed in the INTA S.A. (Unidad de Genómica, Instituto de Biotecnología, Castelar, Argentina) on a 3130xl Genetic Analyzer (Applied Biosystems) with Genescan500(-250)ROX as the internal size standard. AFLP bands between 50 and 500 bp were manually scored with GeneMapper 3.7 (Applied Biosystems). Care was taken to exclude ambiguous loci by checking the peak height frequency distribution for each putative locus and setting an individual peak height cutoff threshold. Markers with a multimodal peak height distribution across samples, potentially indicative of ambiguity, were omitted. Preliminary analyses had revealed that in several populations all individuals had the same multilocus AFLP phenotype across six primer combinations suggesting very high reproducibility and a low error rate.

### Haplotype network and phylogenetic analyses

A statistical parsimony network of haplotypes was constructed using TCS ver. 1.2.1 [58] with the algorithm of Templeton & al. [59]. Network ambiguities were resolved according to the rules based on coalescent theory provided by Pfenninger & Posada [60]. The phylogenetic relationships among cpDNA haplotypes were reconstructed using MrBayes 3.1.2 [61]. ModelTest 0.1.1 [62] was used to identify the model of molecular evolution (GTR+I) that best fit the data matrix under the Akaike information criterion (AIC). Four Monte Carlo Markov chains starting with a random tree were run simultaneously in two independent runs for 10 000 000 generations and trees were sampled every 2000 generations. Sample points collected prior to stationarity (convergence of likelihood scores) were eliminated as burn-in (25%). Posterior probabilities for supported clades were determined by a 50% majority-rule consensus of the retained trees.

### Divergence time

To estimate genetic differentiation among *M*. *argentina* haplotypes during Pliocene, Pleistocene, and Holocene events, we performed molecular dating under a Bayesian approach as implemented in BEAST ver. 2.1.3 [63]. The divergence time of *M*. *argentina*’s haplotypes was estimated using the age obtained from the previous dating for Scleropogoninae and *Swallenia alexandrae* [52–64]. *Sohnsia filifolia* (Muhlenbergiinae, sister clade of Scleropogoninae), *Scleropogon brevifolius*, *Swallenia alexandrae*, *Erioneuron avenaceum*, *Munroa pulchella*, and *Munroa andina* were outgroups. The node of the Scleropogoninae clade was constrained using a lognormal prior distribution (offset: 18.0, Log (mean): 0; Log (Stdev): 0.5, range: 19.5–17.5). The node of the *Swallenia alexandrae* was constrained using a lognormal prior distribution (offset: 16.3, Log (mean): 0; Log (Stdev): 0.5, range: 17.0–15.5). Analyses were run using a molecular clock model with uncorrelated rates, assuming a lognormal distribution of rates. Models of sequence evolution were the same as for the MrBayes analyses (GTR+I) and a coalescent model assuming logistic population growth was selected. Two MCMC analyses were run each with 100 million generations, and sampling every 10 000th generation. The time series plots of all parameters were analyzed in Tracer v.1.5 [65] (http://tree.bio.ed.ac.uk/software/tracer/) to check for adequate effective sample sizes (ESS > 200) and convergence of the model’s likelihood and parameters between each run. Trees were combined in Log Combiner v.1.6.1 [66], setting the burn-in at 25% of the initial samples of each MCMC run. Post-burn-in samples were summarized using the maximum clade credibility tree option in Tree Annotator v.1.6.1 [66].

### Genetic and spatial analyses

For chloroplast data, parameters of population diversity—i.e. number of haplotypes (K), haplotype diversity (*h*; [67]) and nucleotide diversity (*π*; [67])—were calculated for each phylogroup derived from the haplotype network and phylogenetic analyses using Arlequin 3.1 [68]. Genetic variability within and among phylogroups was determined with an analysis of molecular variance (AMOVA) using Arlequin. The number of permutations to determine significance level was set at 10000 replicates. A genetic landscape shape analysis was run using Alleles in Space [69]. This analysis identifies genetic discontinuities among populations in a landscape shape and produces a three-dimensional surface plot where the x and y axes correspond to the geographic coordinates of populations, and the z axis corresponds to the interpolated genetic distances. We also conducted a Mantel test [70] and investigated the genetic barriers associated with each geographic location and each population using Monmonier’s maximum-difference algorithm implemented in Alleles in Space [69].

### Demographic history

Neutrality and mismatch distribution analyses were performed for the phylogroups—identified by TCS, phylogenetic and AMOVA analyses—using Arlequin. Tajima’s *D* [71], Fu’s *Fs* [72] and *R2* [73] were calculated to detect past demographic range expansions using DnaSP v. 5.0 [74]. The significance level of the three values was calculated from 1000 simulated samples using a coalescent algorithm [75]. Mismatch distribution analysis was run to detect either past exponential growth or historical population stasis. The goodness of fit of the observed distribution was evaluated using parametric bootstrapping with the sum of squares deviations. The sum of squares deviations (P ≤ 0.05) analysis indicates a departure from the null model of population expansion. Bayesian skyline plots [76] were obtained using BEAST ver. 2.1.3 [63,77] to describe demographic history by assessing the time variation in effective population size. Two independent runs of 10 million generations were performed using the substitution model GTR with empirical base frequencies, an uncorrelated lognormal relaxed clock model, and a piecewise-constant coalescent Bayesian skyline tree prior with five starting groups. Trees and parameters were sampled every 1000 iterations, with a burn-in of 10%. The time axis was scaled using 1.0–3.0 x 10^−9^ substitutions per site per year (s/s/y) for chloroplast-wide, synonymous substitution rates described for most angiosperms [78], and the rate of 4.5 x 10^−9^ s/s/y calculated for the cpDNA loci in *Munroa* (S1 File). The results of each run were visualized using Tracer to ensure that stationarity and convergence had been reached (ESS > 200).

### AFLP analyses

For nuclear data (AFLP), SplitsTree v. 4.10 [79] was utilized with uncorrected “p” genetic distances and the NeighborNet algorithm to generate a network. The similarity/genetic distance were calculated using the Jaccard similarity measure. A bootstrap analysis was performed with 1 000 replicates. The Bayesian approach implemented in STRUCTURE version 2.3.1 [80,81] was used to analyze the geographic structure of *M*. *argentina* populations. This program uses a coalescent genetic approach to cluster similar multilocus genotypes into K clusters, regardless of an individual’s geographic origin. We conducted five independent runs for each value of K ranging from 1–5 using the no admixture model and correlated allele frequencies [81]. The optimal value of *K* was calculated following the method by Evanno & al. [82]. Genetic variability at several hierarchical levels was determined by an analysis of molecular variance (AMOVA) in Arlequin. The population groups were those recognized by the SplitsTree and Structure analyses. The population parameters such as proportion of polymorphic loci (*P*) and expected heterozygosity (H_E_) were calculated for each population groups using Arlequin and TFPGA v. 1.3 (Tools for Population Genetics Analyses) [83] respectively.

### Ecological Niche Modeling

The ecological niche was estimated based on the available distribution records of *M*. *argentina*. A total of 200 occurrence points were compiled both during the fieldwork of this project and also using specimen records from the following herbaria: CORD, CASTELAR, BAA, BAB, IBODA, LIL, and LPB (Abbreviations for herbaria follow "Index Herbariorum" http://sweetgum.nybg.org/ih/). Environmental scenarios in the present and in the past were represented by a series of 19 variables summarizing aspects of climate [84]; we included all 19 bioclimatic variables because this results in more conservative conclusions about distributional stability that are thus more reliable [42]. Data were obtained from WorldClim 1.4 [84] with a resolution of 1 km^2^. Four estimates of ecological niche models were made to identify: 1) the potential current distribution, 2) the potential past distribution during the Last Interglacial, and 3) the potential past distribution during the extreme conditions during the Last Glacial Maximum (LGM). For the first analysis, we used the current layer, followed by the last interglacial period layer (140,000–120,000 BP), and the LGM (21,000–18,000 BP) layer. For the LGM analysis we used general circulation model simulations from two models: the Community Climate System Model (CCSM; [85]) and the Model for Interdisciplinary Research on Climate (MIROC; [86]). All models were run in MaxEnt and were repeated to obtain 10 replicates. To evaluate the quality of the model, we partitioned the locality data into training and testing data sets (75% and 25%, respectively). To measure the degree to which the models differed from that expected by chance, and to obtain a confidence measure for the ENMs, we used AUC (the area under the receiving operating characteristic curve) [87,88].

## RESULTS

### Phylogenetic analyses and haplotype network

The length of the two combined plastid markers (*rps16-trnK*, *ndhA intron*) was 1704 bp with 43 variable sites. Forty-one haplotypes were identified for the 152 individuals sampled from 20 localities (Fig 1; Table 1 includes the list of the localities). Three phylogroups were identified and are distributed in three different biogeographic provinces: i–Puna (Argentina and Bolivia), ii–Prepuna (Argentina), and iii–Monte (Argentina).

In the Puna phylogroup, the most frequent haplotype, H7, is shared by six localities in both Argentinean and Bolivian Puna (JT, JH, JS, SSC, BY, and BU). Haplotype H5 is shared by three localities BY, BP, BT (Bolivian Puna), and haplotype H13 is common in JT, JH, and JS (Argentinean Puna). Thirteen haplotypes were private to seven localities in both Argentinean and Bolivian Puna (Fig 1 and Table 1). Haplotype H17 is unique to JT and connected the Puna phylogroup with the Prepuna and Monte phylogroups. The Prepuna phylogroup occurs in Argentina and haplotypes H20 and H23 were the most frequent; H20 is shared by two localities, SJJ and SJB, while H23 is unique to MLH. Nine haplotypes were private to four localities (Fig 1 and Table 1). The Prepuna and Monte phylogroups are connected by haplotypes H18 and H29; the latter are private to LRF. In the Monte phylogroup that occurs in Argentina, haplotype H30 is the most frequent and is shared by three localities: CCQ, CB, and TAV. Finally, eleven haplotypes were private to five localities in Argentinean Monte (Fig 1 and Table 1).

**Fig 2:**
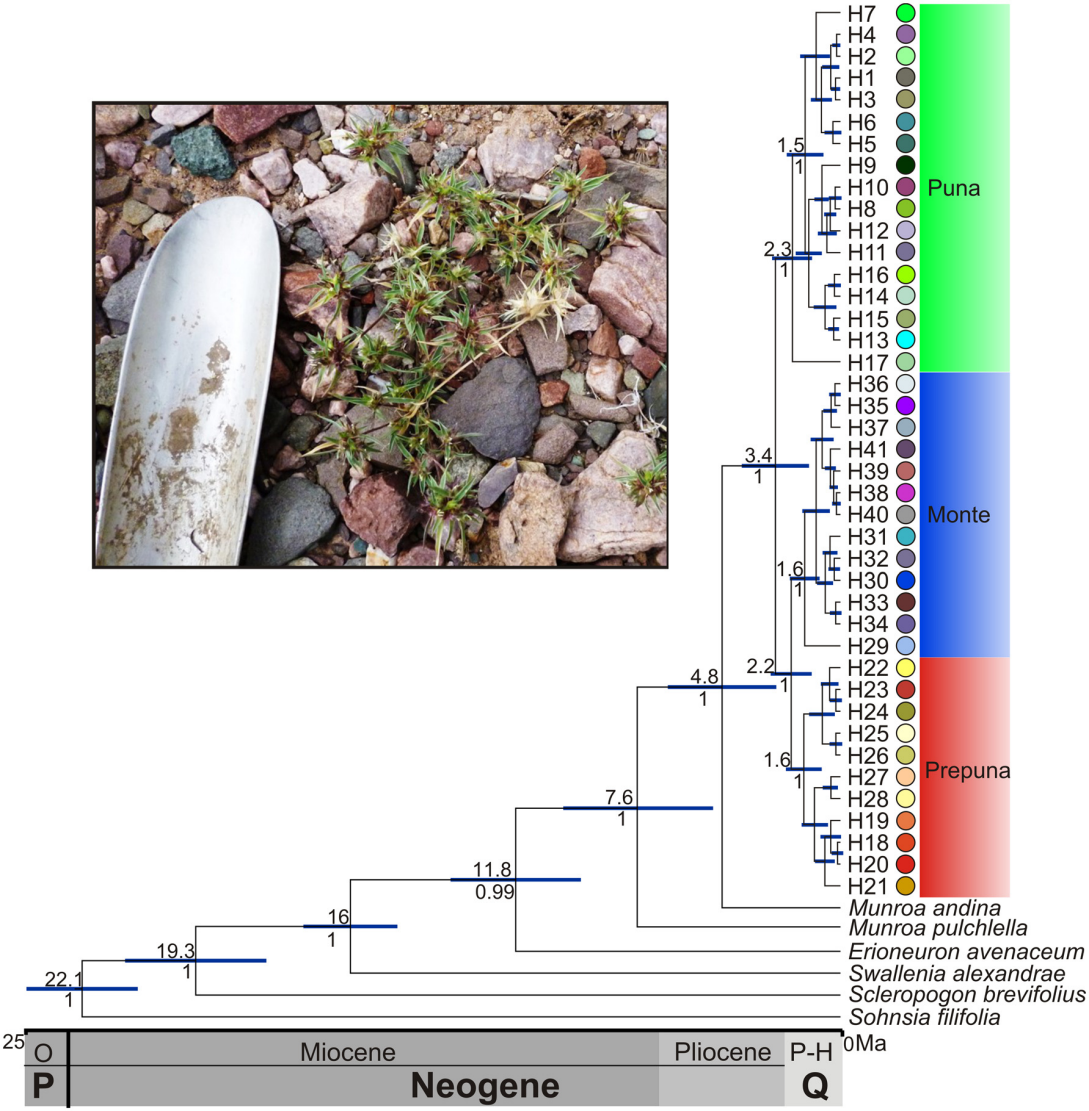
Maximum clade credibility tree of the cpDNA haplotypes generated from BEAST. Chronogram of *M*. *argentina* haplotypes and other Chloridoideae based on the consensus tree from the Bayesian dating analysis using a coalescent model with a constant size. All nodes are provided with the 95% Highest Posterior Density intervals (purple bars), the time of divergence (above branch) and the posterior probabilities (below branch). The individual haplotype and haplotype group designations correspond to those in Fig 1. Inset photo is *M*. *argentina* in San Antonio de los Cobres, Salta, Argentina. Photograph by Vanesa Pilatti.

The 50% majority consensus tree (results not shown) grouped all *M*. *argentina* haplotypes in a single and well supported clade (PP = 1.00), corroborating the monophyly of *M*. *argentina*. The topology of the recovered phylogram (not shown) is congruent with the result of the statistical parsimony network and identical to the chronogram obtained with BEAST (see below, Fig 2). Three major clades were identified; the first formed by the Puna phylogroup and the other two by the Prepuna and Monte phylogroups.

### Divergence time

According to our molecular dating, the diversification of phylogroups of *M*. *argentina* began in the Middle Pliocene–Late Pleistocene, approximately 3.4 (4.2–1.2) Ma separating the Puna phylogroup from the ancestor of the Prepuna–Monte phylogroups (Fig 2). The Prepuna phylogroup diverged from the Monte phylogroup approximately 2.2 (3.0–1.1) Ma during the Pleistocene. The diversification of the majority of the haplotypes began 1.6 (1.9–0.6) Ma in the Mid-Pleistocene (Fig 2).

### Genetic and spatial analyses

**Fig 3:**
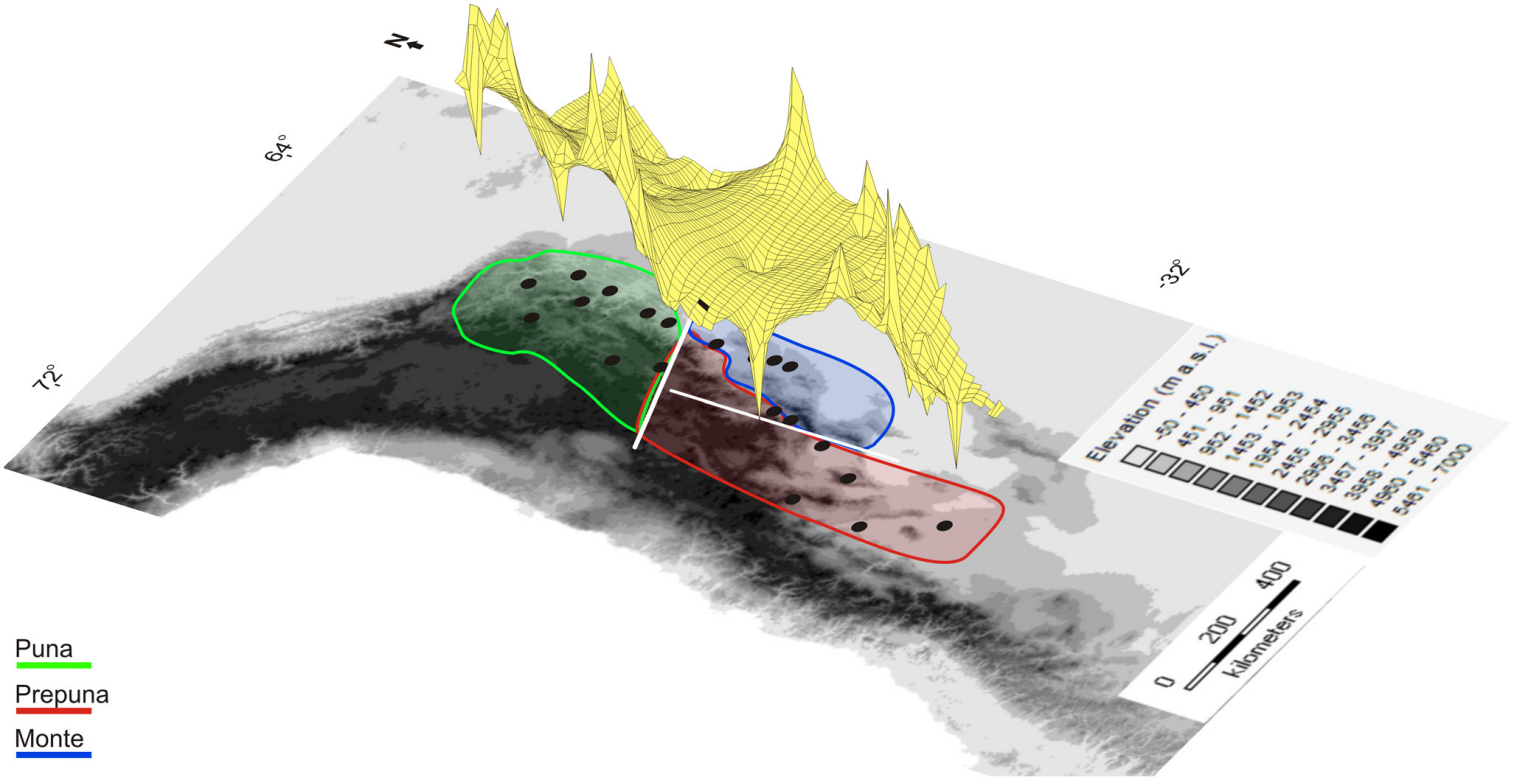
Multidimensional graph produced by the genetic landscape shape interpolation analysis that represents the genetic distances of haplotypes across the distribution range of *M*. *argentina*. White bars identify phylogeographic barriers in the unglaciated part of the SATZ that separate three groups of populations: Puna, Prepuna and Monte.

The phylogroup haplotype diversity (h) ranged 0.78–0.85, while nucleotide diversity (π) ranged 0.0008–0.0012 (Table 2). AMOVA revealed strong population structure, with the highest F_ST_ value obtained when samples were grouped in three groups: Prepuna, Puna and Monte (F_ST_ = 0.77; Table 3). The Mantel test (r = 0.51; P = 0.001) revealed a significant relationship between geographical and genetic distances and supported the hypothesis of isolation by distance. Monmonier’s algorithm also revealed strong differentiation among the northern phylogroups. Two major genetic barriers were identified: one, separating Puna and Prepuna from Monte phylogroups, and the other separating Puna from Prepuna and Monte phylogroups (Fig 3). The results of the genetic landscape analysis were congruent with these results, with three clearly differentiated zones: the first, at the extreme north of this species distribution, included the Puna region localities, the second was smaller and included the Monte region localities at the eastern margin of the range, and the third included the Prepuna region localities at the southern margin of the range (Fig 3).

**Table 2:** Results of genetic and demographic analyses including probability of capturing the deepest coalescent event (Prob.), number of haplotypes (*K*), haplotype diversity (*h*), nucleotide diversity (π), Fu’s *Fs* (*Fs*), Tajima’s D (D_T_), Probability of D_T_ ≠ 0 [Prob. (|D_T_|) > 0], probability of D_T_ ≠ 0 based on coalescent simulations (P), Ramos-Onsins & Rozas’ (*R2*), probability of *R2* based on coalescent simulations (*P*), maximum pairwise differences between any two sequences (*k*).

Population	n	Prob.	*K*	*h* (± SD)	*π* (± SD)	*Fs*/D_T_	Prob.(|D_T_|)>0/P	*R2*/*P*/*k*	SSD
**Puna**	59	0.96	17	0.80 (± 0.04)	0.0012 (± 0.0008)	-8.19/-1.59	|-1.6|/0.03	0.049/0.018/2.1	0.00277
**Prepuna**	47	0.95	12	0.85 (± 0.03)	0.0010 (± 0.0007)	-1.75/-0.40	|-0.4|/0.41	0.095/0.361/1.8	0.02329
**Monte**	46	0.95	12	0.78 (± 0.06)	0.0008 (± 0.0006)	-7.51/-1.59	|-1.6|/0.03	0.052/0.012/1.5	0.00132
**Total**	152	0.98	41	0.92 (± 0.01)	0.0021 (± 0.0001)	-8.31/-1.55	|-1.5|/0.02	0.041/0.021/3.7	0.00235

The sum of squared deviations (SSD) is also given.

**Table 3:** Structure of variation in *M*. *argentina* analyzed using an AMOVA with alternative groupings.

Source of variation	d.f.	Percentage of variation	*F-*statistic
**cpDNA data**.			
**a) three groups according to haplotype network and Bayesian inference, Puna vs. Prepuna vs. Monte**			
among groups	2	45.1	
among populations within groups	1	31.8	FCT = 0.45***
within populations	148	23.1	F_SC_ = 0.57***
Total	151		F_ST_ = 0.77***
**b) two groups, Puna/Prepuna vs. Monte**			
among groups	1	7.4	
among populations within groups	1	66.9	F_CT_ = 0.07***
within populations	149	25.6	F_SC_ = 0.72***
Total	151		F_ST_ = 0.74***
**c) two groups, Puna/Monte vs. Prepuna**			
among groups	1	2.5	
among populations within groups	1	68.6	F_CT_ = 0.02***
within populations	149	28.9	F_SC_ = 0.76***
Total	151		F_ST_ = 0.70***
**d) two groups, Puna vs. Monte/Prepuna**			
among groups	1	18.8	
among populations within groups	1		F_CT_ = 0.18***
within populations	149		F_SC_ = 0.69***
Total	151	56.1	F_ST_ = 0.74***
**AFLP data**		24.9	
**a) three groups according to Split Tree and Structure, Puna vs. Monte vs. Prepuna**			
among groups	2	25.1	
among populations within groups	1	1.5	F_CT_ = 0.25***
within populations	148	73.4	F_SC_ = 0.02***
Total	151		F_ST_ = 0.27***

d.f., degrees of freedom; F_CT_, differentiation among groups within the species; F_SC_, differentiation among populations within groups; F_ST_, differentiation among populations within the species.

***P < 0.0001.

### Demographic history

Under a model of sudden population expansion (P>0.05), the Puna and Monte phylogroups have a strongly unimodal mismatch distribution indicating a historical population expansion event, while the Prepuna phylogroup have a bimodal mismatch distribution, indicating a historical population contraction/expansion (S1 Fig). The same unimodal mismatch distribution pattern is found when all of the haplotypes were included. Fu’s *Fs*, Tajima’s *D*, *R2* test, and the SSD values suggest a historical population expansion by three phylogroups defined by TCS and phylogenetic analyses (Table 2). The Bayesian skyline plots reveal similar demographic histories among the three phylogroups analyzed (S1 Fig). The Puna phylogroup may have increased over the past 90,000 years, the Prepuna phylogroup had a stable population size over the last 60,000 years, and the Monte phylogroup may have increased over the past 85,000 years. Estimates for the latter two phylogroups had larger variances as a consequence of smaller sample sizes. The Prepuna+Monte phylogroups would have begun to expand 80,000 years ago.

### AFLP analyses

**Fig 4:**
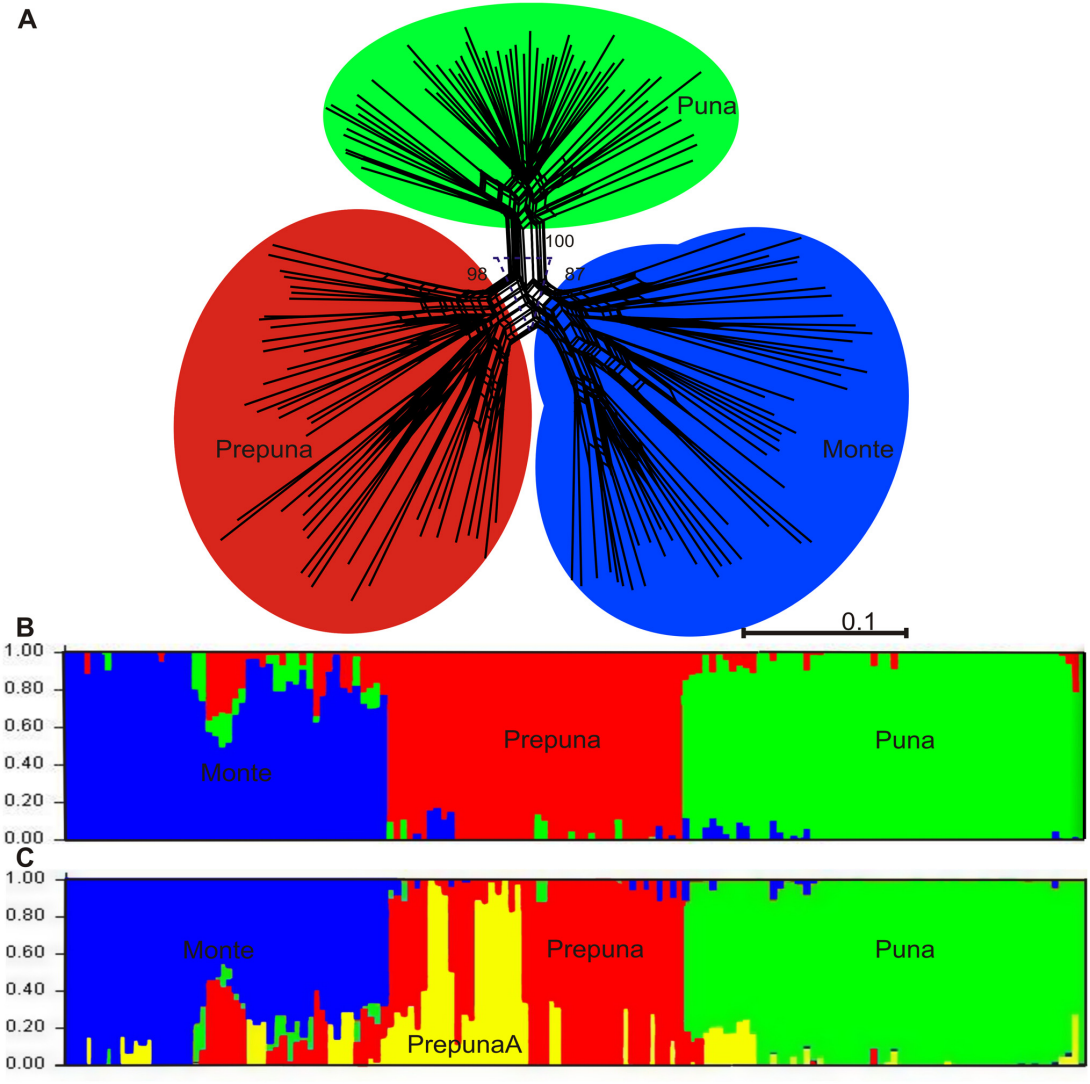
Inferred clustering from NeighborNet (A) (numbers are the bootstrap values), and from a Bayesian assignment with STRUCTURE assuming *K* = 3 (B), and *K* = 4 (C). Each individual is represented by a single vertical line, partitioned into *K* colored segments that denote the individual’s estimated membership fractions in *K* clusters. Scale bar shows a distance of 0.1 substitutions per site.

In total 152 individuals from 20 localities were genotyped using six primer combinations, 1104 loci were scored, 1075 of which (97%) were polymorphic. The neighbor-net diagram showed a genetic split between three groups with a high degree of support (Bootstrap support = 87–100). The three groups were identical to groups recognized by the haplotype network and the phylogenetic analyses (Puna, Prepuna, and Monte) (Fig 4A). Bayesian clustering analyses implemented in Structure corroborated cluster (phylogroup) structure and the results of *ΔK* indicated that the most likely number of population clusters was three (K = 3) and four (K = 4) (Fig 4B and 4C, respectively). With K = 3, the classification of individuals into groups fully corresponded to the haplotype network, phylogenetic and SplitsTree analyses. With K = 4, the structure of the three population clusters was constant, but a subgroup (Prepuna) appeared for the Prepuna population clusters. The AMOVA—with the three population clusters recognized by the Splits Tree and the Structure (K = 3) analysis for AFLP data, and Bayesian inference for cpDNA data—revealed that most of the genetic variation was within populations (73.4%), while variation among clusters and among populations within clusters was 25.1% and 1.5%, respectively. The Prepuna phylogroup expected heterozygosity (He) and polymorphism (P) were the highest among the three phylogroups (0.31 and 88.5% vs. 0.28 and 63.1%, Puna; 0.19 and 77.4%, Monte).

### Niche-based distribution modeling

**Fig 5:**
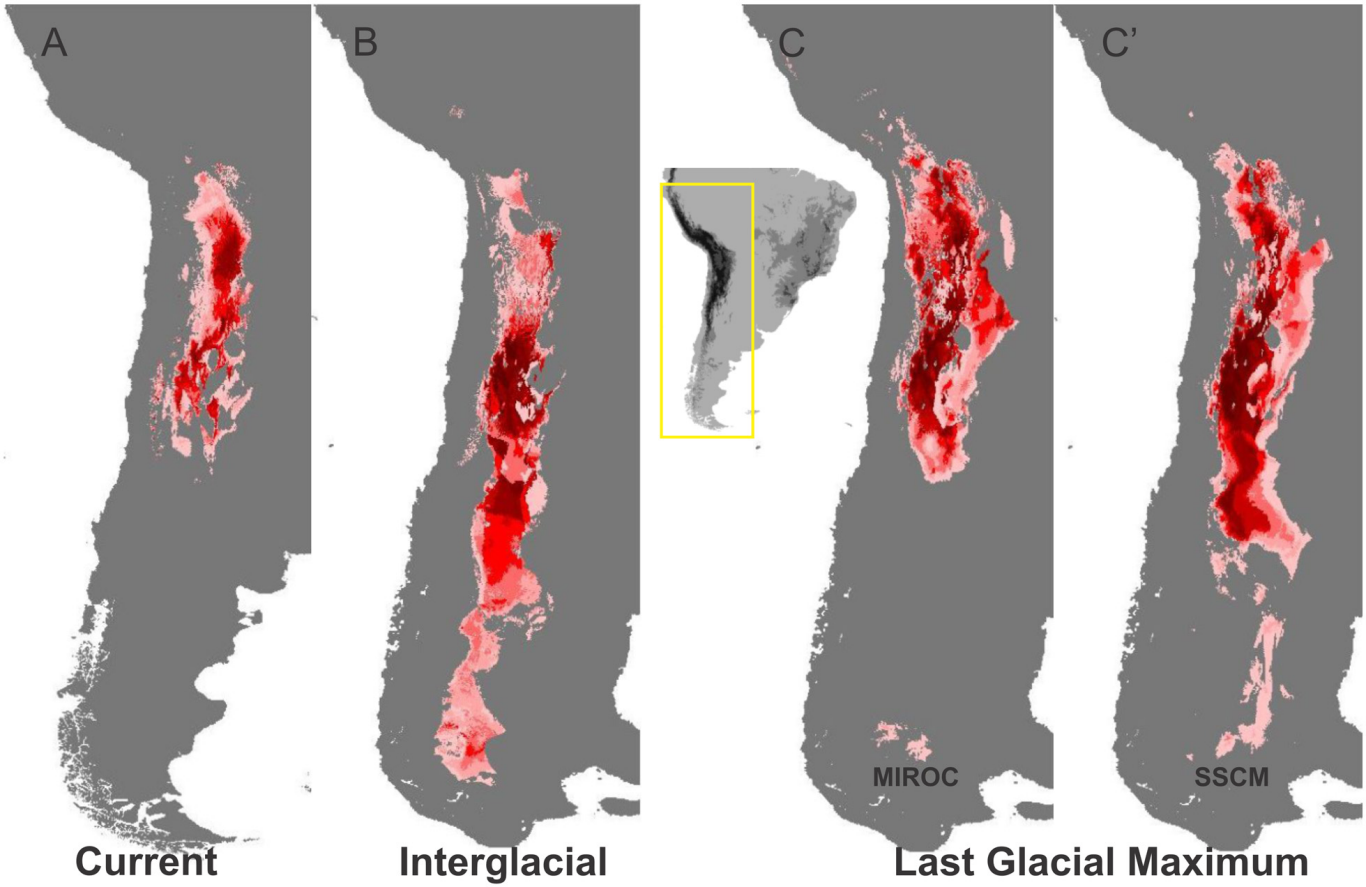
Climate-based predicted distribution of *M*. *argentina* for three geological time periods. Predicted distribution during the Last Glacial Maximum was obtained using MIROC and CCSM models.

The value of AUC was > 0.95 for all analyses. Niche distribution modeling for current climate conditions over-predicts the geographic distribution in the extreme north and southeast of *M*. *argentina* where it has never been recorded (Fig 5A and 5B). The potential distribution during the Last Interglacial Period was continuous and covered a more extensive area of suitable habitats from northern Prepuna province to the Patagonia Subregion (Andean Region) (Fig 5B). The Last Glacial Maximum model indicates that suitable habitats were more extensive than they currently are (and less extensive than they were during the Interglacial), and over-predicts the geographic distribution further to the southeast (Fig 5C–5C’).

## DISCUSSION

### Phylogeography

Geological, climate, and genetic studies suggest that the SATZ arose from the Middle Miocene to the Upper Pliocene [7,19,20,29,33], and indicate the persistence of a semiarid climate between 8 and 3 Ma that reached its highest aridity level about 6 Ma [29]. The split between *M*. *argentina* and the ancestor shared with *M*. *andina* occurred 4.8 (5.2–2.9) Ma from the Late Miocene to the Late Pliocene when climate conditions were suitable, which agrees with our previous results [52]. Analyses of cpDNA sequences and AFLP data revealed three highly divergent lineages within *M*. *argentina*. The split among these phylogroups occurred in the Late Pliocene (4.2–1.2 Ma) and mainly in the Pleistocene. These results suggest that although the early stages of the evolutionary history of *M*. *argentina* (i.e. its divergence) are linked to the (semi)aridification processes that gave rise to the SATZ, and to the final uplift of the Southern Andes (5 Ma), the following stages and conformation of its range are rather linked to Quaternary glaciations (1 Ma– 15 Ka) [7,20,29,44,89,90]. Climate oscillations in the Pleistocene are probably among the causes of strong differentiation among phylogroups of *M*. *argentina*, through the fragmentation of an ancient, more widespread distribution. The three phylogroups (Puna, Prepuna and Monte) do not share any haplotypes; moreover, the phylogroups identified by cpDNA and AFLP data were congruent. These results are consistent with a hypothesis of ancient habitat fragmentation, as previously posited by Templeton [91] and reported for species endemic to desert areas and the central area of Patagonia [33,42,44].

It is commonly inferred that areas exhibiting high levels of genetic variation were glacial refugia for relatively large populations [92], while areas with low genetic variation are interpreted as recently colonized or harboring small relict populations [37,42,93]. In *M*. *argentina*, although the highest within-population genetic variation was found in the southern area of the Prepuna, genetic variation was also high in Prepuna and Monte. Prepuna is the southern limit of this species’ distribution, suggesting that it was probably the habitat occupied by the ancestors of *M*. *argentina*. Demographic parameters, the negative value of Fu’s *Fs* and Tajima’s *D*, and the results of the mismatch distribution analysis all suggest a growing population size in *M*. *argentina*. Based on this evidence, we suggest that populations extended their range northward and westward into the Puna and Monte provinces, respectively. From there, the once continuous distribution underwent fragmentation due to the climatic oscillations in the Pliocene-Pleistocene.

Our results show significant phylogeographic structure within the three areas in the SATZ; this structure is evident in the haplotype network, phylogenetic tree and high Fst values. Although the range of distribution of *M*. *argentina* appears to be continuous, its populations are actually isolated. From our field observations, we noticed that while the populations are often found within short distances of each other, they inhabit areas with different ecological conditions and their flowering periods differ considerably (Table 1). The Puna phylogroup from eastern Bolivia, northern Argentina, and southern Peru reach the highest elevations—from 2800 to 4200 m a.s.l.—and the plants bloom from mid-February to April. The Prepuna phylogroup from central and northwestern Argentina grow at mid-elevations from 1500 to 2900 m a.s.l. and the plants bloom from mid-December to February. The Monte phylogroup from central Argentina grows at the lowest elevations, from 1000 to 2500 m a.s.l. and the plants bloom from February to March. Similar differentiation has been reported for several species of *Hordeum* in the SATZ [39,42], and differences in ecological conditions have been suggested as a mechanism responsible for the isolation of phylogroups [39]. Prezygotic isolation caused by differences in flowering time might further limit interpopulation gene flow, and increase the isolation of lineages caused by initial habitat discontinuity.

### Pleistocene and present distribution areas

Ecological niche modeling has been applied to a variety of research topics, including speciation mechanisms [94], species extinction [94], niche shifts [15,39,42], and paleomodels, with the goal of improving phylogeographic inference [95]. Both the AFLP and cpDNA data suggest that *M*. *argentina* not only retained its distribution from the Pliocene until the present time, but that it also extended northward to warmer areas during the last of the Pleistocene cold cycles, and this was confirmed by the niche modeling. Current modeling and paleomodeling were applied to analyze the suitability of present climate and conditions during glacial maxima and interglacial periods for *M*. *argentina*. For the current climate, the predicted area of suitable habitats includes not only the southeast where this species grows now but also east of the Monte province, where *M*. *argentina* has not yet been found. This region has more humid conditions than the northern areas of the Monte and Prepuna provinces, and its vegetation consists of taller grasses, which might prevent colonization by *M*. *argentina*. This may explain why the species is not distributed in these areas even though climate conditions are suitable for *M*. *argentina*.

The predicted suitable range of *M*. *argentina* during the LGM and interglacial much exceeded its present range and also included areas north, south, and east of the currently occupied areas. The predicted suitable range of *M*. *argentina* during the interglacial period includes zones in the south reaching the Central Patagonia province. These results suggest that climate conditions during the Interglacial periods and the LGM did not limit the expansion of *M*. *argentina* on the eastern side of the SATZ. They also support our hypothesis about the effects of habitat fragmentation on populations (Prepuna, Puna, Monte), given that large suitable areas are necessary for population subdivisions to occur without any marked reduction in genetic diversity; similar results were found for *Hordeum* [42].

Our study utilized two independent approaches to reconstruct the present and past population distribution. The phylogeographic approach detected survival of *M*. *argentina* within its extant distribution for at least the last ice age cycle. The ecological niche modeling, revealed that the climate conditions were suitable for *M*. *argentina* in the SATZ, and that the niche for this species has not changed since at least the LGM.

Recent studies are finding complex phylogeographic patterns in the biota of South America [7,15,41–42,95–99] and although the Andean uplift was one of the major events with a clear effect on the diversification of South American biota [1–3,7,9,41,96–100], climate change in the Late Neogene and associated glaciations significantly contributed to the regional plant evolution and diversity[1–3,7,9,41,96–100].

## Conclusions

Our study has revealed that, since approximately 4 Ma, the grass species *Munroa argentina* has been able to persist in the SATZ despite orogenic changes, climate changes and ice age cycles, and that its apparently once continuous range has undergone fragmentation. Our analyses detected a deep intraspecific phylogeographic structure with multiple lineages for this species. We identified three phylogroups (Puna, Prepuna, Monte) with low historical gene flow among them and a strong genetic structure that matches geographic distributions in known biogeographic provinces with distinct histories.

## Supporting Information

S1 Fig Mismatch distributions and Bayesian skyline plots of cpDNA haplotypes. **A**-**D**, Mismatch distributions of pairwise nucleotide differences for population clusters of *M*. *argentina*. Dashed lines show the observed frequency distributions and solid lines show the distribution expected under the sudden-expansion model. **E**-**I**, Bayesian skyline semilog plots showing medians for the historical demographic trends for total populations of *M*. *argentina*.
(TIF)

S1 Table Haplotype number (H) for *M*. *argentina*, the outgroup taxa sampled, and GenBank accession numbers (the first *ndhA intron*, the second *rps16-trnK*). (DOCX)

S1 File Demographic history methods. (DOCX)

## References

[1] Taylor DW (1991) Paleobiogeographic relationships of Andean angiosperms of Cretaceous to Pliocene age. Palaeogeography, Palaeoclimatology, Palaeoecology 88: 69–84.

[2] Hoorn C (1993) Marine incursions and the influence of Andean tectonics on the Miocene depositional history of northwestern Amazonia: Results of a palynostratigraphic study. Palaeogeography, Palaeoclimatology, Palaeoecology 105: 267–309.

[3] Hoorn C, Guerrero J, Sarmiento GA, Lorente MA (1995) Andean tectonics as a cause of changing drainage patterns in Miocene northern South America. Geology 23: 237–240.

[4] Gregory-Wodzicki KM (2000) Uplift history of the central and northern Andes: A review. Geological Society of America Bulletin 112: 1091–1105.

[5] Garzione CN, Hoke GD, Libarkin JC, Withers S, MacFadden B, Eiler J, Ghosh P, Mulch A (2008) Rise of the Andes. Science 320: 1304–1307. 10.1126/science.1148615 18535236

[6] Ramos VA, Ghiglione MC (2008) Tectonic evolution of the Patagonian Andes In: Rabassa J, editor. The late Cenozoic of Patagonia and Tierra del Fuego. Elsevier, Oxford pp. 205–226.

[7] Guerrero PC, Rosas M, Arroyo MTK, Wiens JJ (2013) Evolutionary lag times and recent origin of the biota of an ancient desert (Atacama–Sechura). Proceedings of the National Academy of Sciences USA 110: 11469–11474. 10.1073/pnas.1308721110 PMC3710863 23798420

[8] Palma RE, Marquet PA, Boric-Bargetto D (2005) Inter- and intraspecific phylogeography of small mammals in the Atacama Desert and adjacent areas of northern Chile. Journal of Biogeography 32: 1931–1941.

[9] Antonelli A, Nylander JAA, Perssona C, Sanmartín I (2009). Tracing the impact of the Andean uplift on Neotropical plant evolution. Proceedings of the National Academy of Sciences USA 106: 9749–9754. 10.1073/pnas.0811421106 PMC2685738 19470489

[10] von Hagen KB, Kadereit JW (2003) The diversification of *Halenia* (Gentianaceae): Ecological opportunity versus key innovation. Evolution 57: 2507–2518. 14686527

[11] Bell CD, Donoghue MJ (2005) Phylogeny and biogeography of Valerianaceae (Dipsacales) with special reference to the South American valerians. Organisms Diversity & Evolution 5: 147–159.

[12] Winkworth RC, Donoghue MJ (2005) *Viburnum* phylogeny based on combined molecular data: Implications for taxonomy and biogeography. American Journal of Botany 92: 653–666. 10.3732/ajb.92.4.653 21652443

[13] Hughes C, Eastwood R (2006) Island radiation on a continental scale: Exceptional rates of plant diversification after uplift of the Andes. Proceedings of the National Academy of Sciences USA 103: 10334–10339. 10.1073/pnas.0601928103 PMC1502458 16801546

[14] Pirie MD, Chatrou LW, Mols JB, Erkens RHJ, Oosterhof J (2006) Andean-centred genera in the short-branch clade of Annonaceae: Testing biogeographical hypothesis using phylogeny reconstruction and molecular dating. Journal of Biogeography 33: 31–46.

[15] Jakob SS, Heibl C, Rödder D, Blattner FR (2010) Population demography influences climatic niche evolution: evidence from diploid American *Hordeum* species (Poaceae). Molecular Ecology 19: 1423–1438. 10.1111/j.1365-294X.2010.04582.x 20456231

[16] Popp M, Mirré V, Brochmann C (2011) A single Mid-Pleistocene long-distance dispersal by a bird can explain the extreme bipolar disjunction in crowberries (*Empetrum*). Proceedings of the National Academy of Sciences USA 108: 6520–6525. 10.1073/pnas.1012249108 PMC3081031 21402939

[17] González F, Wagner ST, Salomo K, Symmank L, Samain M, Isnard S, Rowe NP, Neinhuis C, Wanke S (2014) Present trans-Pacific disjunct distribution of *Aristolochia* subgenus *Isotrema* (Aristolochiaceae) was shaped by dispersal, vicariance and extinction. Journal of Biogeography 41: 380–391.

[18] Ribas CC, Moyle RG, Miyaki CY, Cracraft J (2007) The assembly of montane biotas: linking Andean tectonics and climatic oscillations to independent regimes of diversification in *Pionus* parrots. Proceedings of the Royal Society B: Biological Sciences 274: 2399–2408. 17686731 10.1098/rspb.2007.0613 PMC2274971

[19] Le Quesne C, Acuna C, Boninsegna JA, Rivera A, Barichivich J (2008) Long-term glacier variations in the Central Andes of Argentina and Chile, inferred from historical records and tree-ring reconstructed precipitation. Palaeogeography, Palaeoclimatology, Palaeoecology 281: 334–344.

[20] Hoorn C, Wesselingh FP, ter Steege H et al (2010) Amazonia through time: Andean uplift, climate change, landscape evolution and biodiversity. Science 330:927–993. 10.1126/science.1194585 21071659

[21] Cabrera AL, Willink A (1980) Biogeografía de América Latina O.E.A. Serie de Bioloía, Monografía 13. Ed. 2, corr. General Secretariat of the Organization of American States, Washington, DC.

[22] Morrone JJ (2006) Biogeographic areas and the transition zone of Latin America and the Caribbean Islands based on panbiogeographic and cladistics analyses of the entomofauna. Annual Review of Entomology 51: 467–494. 16332220 10.1146/annurev.ento.50.071803.130447

[23] Arroyo MTK, Squeo FA, Armesto JJ, Villagrán C (1988) Effects of aridity on plant diversity in the northern Chilean Andes: Results of a natural experiment. Annals of the Missouri Botanical Garden 75: 55–78.

[24] Schulz N, Boisier JP, Aceituno P (2012) Climate change along the arid coast of northern Chile. International Journal of Climatology 32: 1803–1814.

[25] Morrone JJ (2013) Cladistic biogeography of the Neotropical region: identifying the main events in the diversification of the terrestrial biota. Cladistics 30: 202 214. 10.1111/cla.12039 34784690

[26] Comes HP, Kadereit JW (1998) The effect of Quaternary climatic changes on plant distribution and evolution. Trends in Plant Science 3: 432–438.

[27] Hewitt GM (2000) The genetic legacy of the Quaternary ice ages. Nature 405: 907–913. 10879524 10.1038/35016000

[28] Jackson ST, Overpeck JT (2000) Responses of plant populations and communities to environmental changes of the late Quaternary. Paleobiology 26: 194–220.

[29] Hartley AJ, Chong G (2002) Late Pliocene age for the Atacama Desert: Implications for the desertification of western South America. Geology 30: 43–46.

[30] Williams JW, Shuman BN, Webb T, Bartlein PJ, Leduc PL (2004) Late-Quaternary vegetation dynamics in North America: scaling from taxa to biomes. Ecological Monographs 74: 309–334.

[31] Bouchenak-Khelladi Y, Verboom GA, Savolainen V, Hodkinson TR (2010) Biogeography of the grasses (Poaceae): A phylogenetic approach to reveal evolutionary history in geographical space and geological time. Botanical Journal of the Linnean Society 162: 543–557.

[32] Edwards EJ, Osborne CP, Strömberg CAE, Smith SA (2010) The origins of C_4_ grasslands: Integrating evolutionary and ecosystem science. Science 328: 587–591. 10.1126/science.1177216 20431008

[33] Viruel J, Catalán P, Segarra-Moragues JG (2012) Disrupted phylogeographical microsatellite and chloroplast DNA patterns indicate a vicariance rather than long-distance dispersal origin for the disjunct distribution of the Chilean endemic *Dioscorea biloba* (Dioscoreaceae) around the Atacama Desert. Journal of Biogeography 39:1073–1085.

[34] Avise JC (2000) Phylogeography. Harvard University Press 464 p.

[35] Abbott RJ, Smith LC, Milne RI, Crawford RMM, Wolff K, Balfour J (2000) Molecular analysis of plant migration and refugia in the Arctic. Science 289: 1343–1346. 10958779 10.1126/science.289.5483.1343

[36] Kadereit JW, Griebeler EM, Comes HP (2004) Quaternary diversification in European alpine plants: Pattern and process. Philosophical Transactions of the Royal Society of London. Series B: Biological Sciences 359: 265–274. 10.1098/rstb.2003.1389 PMC1693311 15101582

[37] Koch MA, Kiefer C, Ehrich D, Vogel J, Brochmann C, Mummenhoff K (2006) Three times out of Asia Minor: The phylogeography of *Arabis alpina* L. (Brassicaceae). Molecular Ecology 15: 825–839. 16499705 10.1111/j.1365-294X.2005.02848.x

[38] Alsos IG, Eidesen PB, Ehrich D, Skrede I, Westergaard K, Jacobsen GH, Landvik JY, Taberlet P, Brochmann C (2007) Frequent long-distance plant colonization in the changing Arctic. Science 316: 1606–1609. 17569861 10.1126/science.1139178

[39] Jakob SS, Ihlow A, Blattner FR (2006) A chloroplast genealogy of *Hordeum* (Poaceae): long-term persisting haplotypes, incomplete lineage sorting, regional extinction, and the consequences for phylogenetic inference. Molecular Biology and Evolution 23: 1602–1612. 16754643 10.1093/molbev/msl018

[40] Soltis DE, Morris AB, McLachlan JS, Manos PS, Soltis PS (2006) Comparative phylogeography of unglaciated eastern North America. Molecular Ecology 15: 4261–4293. 17107465 10.1111/j.1365-294X.2006.03061.x

[41] Turchetto-Zolet AC, Pinheiro F, Salgueiro F, Palma-Silva C (2013) Phylogeographical patterns shed light on evolutionary process in South America. Molecular Ecology 22: 1193–1213. 10.1111/mec.12164 23279129

[42] Jakob SS, Martinez-Meyer E, Blattner FR (2009) Phylogeographic analyses and paleodistribution modeling indicate Pleistocene in situ survival of *Hordeum* species (Poaceae) in southern Patagonia without genetic or spatial restriction. Molecular Biology and Evolution 26: 907–923. 10.1093/molbev/msp012 19168565

[43] Ossa PG, Perez F, Arnesto JJ (2013) Phylogeography of two closely related species of *Nolana* from the coastal Atacama Desert of Chile: Post-glacial population expansions in response to climate fluctuations. Journal of Biogeography 40: 2191–2203.

[44] Baranzelli MC, Johnson LA, Cosacov A, Sérsic AN (2014) Historical and ecological divergence among populations of *Monttea chilensis* (Plantaginaceae), an endemic endangered shrub bordering the Atacama Desert, Chile. Evolutionary Ecology 28: 751–774.

[45] Hollin JT, Schilling DH (1981) Late Wisconsin–Weichselian mountain glaciers and small ice caps In: Denton GH, Hughes TJ, editors. The last great ice sheets. New York: John Wiley and Sons pp. 179–206.

[46] Rabassa J, Clapperton CM (1990) Quaternary glaciations of the southern Andes. Quaternary Science Reviews 9: 153–174.

[47] Hulton NRJ, Purves RS, McCulloch RD, Sugden DE, Bentley MJ (2002) The last glacial maximum and deglaciation in southern South America. Quaternary Science Reviews 21: 233–241.

[48] Luebert F, Wen J (2008) Phylogenetic analysis and evolutionary diversification of *Heliotropium sect*. *Cochranea* (Heliotropiaceae) in the Atacama Desert. Systematic Botany 33: 390–402.

[49] Schmidt-Jabaily R, Sytsma KJ (2010) Phylogenetics of *Puya* (Bromeliaceae): Placement, major lineages, and evolution of Chilean species. American Journal of Botany 97: 337–356. 10.3732/ajb.0900107 21622394

[50] Romanutti AA, Amarilla LD, Anton AM (2012) *Munroa* In: Zuloaga F, Anton AM, editors. Flora Argentina. Plantas vasculares de la República Argentina. Poaceae. Córdoba, Argentina: Gráficamente Ediciones, 3(I): pp.154–157.

[51] Amarilla LD, Chiapella JO, Nagahama N, Anton AM (2013) Inclusion of *Dasyochloa* in the amphitropical genus *Munroa* (Poaceae, Chloridoideae) based on morphological evidence. Darwiniana, nueva serie 1: 241–252.

[52] Amarilla LD, Chiapella JO, Sosa V, Moreno NC, Anton AM (In press) A tale of North and South America: Time and mode of dispersal of the genus amphitropical *Munroa* (Poaceae, Chloridoideae). Botanical Journal of the Linnean Society.

[53] Doyle JJ, Doyle JA (1987) A rapid DNA isolation procedure for small quantities of fresh leaf tissue. Phytochemistry Bulletin 19: 11–15.

[54] Peterson PM, Romaschenko K, Johnson G (2010) A classification of the Chloridoideae (Poaceae) based on multi-gene phylogenetic trees. Molecular Phylogenetics and Evolution 55: 580–598. 10.1016/j.ympev.2010.01.018 20096795

[55] Tamura K, Peterson D, Peterson N, Stecher G, Nei M, Kumar S (2011) MEGA5: Molecular evolutionary genetics analysis using maximum likelihood, evolutionary distance, and maximum parsimony methods. Molecular Biology and Evolution 28: 2731–2739. 10.1093/molbev/msr121 21546353 PMC3203626

[56] Edgar RC (2004) MUSCLE: A multiple sequence alignment method with reduced time and space complexity. BMC Bioinformatics 5: 113 15318951 10.1186/1471-2105-5-113 PMC517706

[57] Lachmuth S, Durka W, Schurr F (2010) The making of a rapid plant invader: genetic diversity and differentiation in the native and invaded range of *Senecio inaequidens* . Molecular Ecology 19: 3952–3967. 10.1111/j.1365-294X.2010.04797.x 20854275

[58] Clement M, Posada D, Crandall KA (2000) TCS: A computer program to estimate gene genealogies. Molecular Ecology 9: 1657–1659. 11050560 10.1046/j.1365-294x.2000.01020.x

[59] Templeton AR, Crandall KA, Sing CF (1992) A cladistic analysis of phenotypic associations with haplotypes inferred from restriction endonuclease mapping and DNA sequence data. III. Cladogram estimation. Genetics 132: 619–633. 1385266 10.1093/genetics/132.2.619 PMC1205162

[60] Pfenninger M, Posada D (2002) Phylogeographic history of the land snail *Candidula unifasciata* (Helicellinae, Stylommatophora): Fragmentation, corridor migration, and secondary contact. Evolution 56: 1776–1788. 12389722 10.1111/j.0014-3820.2002.tb00191.x

[61] Ronquist F, Huelsenbeck JP (2003) MrBayes 3: Bayesian phylogenetic inference under mixed models. Bioinformatics 19: 1572–1574. 12912839 10.1093/bioinformatics/btg180

[62] Posada D (2008) jModelTest: Phylogenetic model averaging. Molecular Biology and Evolution 25: 1253–1256. 10.1093/molbev/msn083 18397919

[63] Bouckaert R, Heled J, Kühnert D, Vaughan T, Wu C-H, Xie D, Suchard MA, Rambaut A, Drummond AJ (2014) BEAST 2: A Software Platform for Bayesian Evolutionary Analysis. PLoS Computational Biology, 10(4), e1003537 10.1371/journal.pcbi.1003537 24722319 PMC3985171

[64] Bouchenak-Khelladi Y, Verboom GA, Hodkinson TR, Salamin N, Francois O, Chonghaile GN, Savolainen V (2009) The origins and diversification of C_4_ grasses and savanna-adapted ungulates. Global Change Biology 15: 2397–2417.

[65] Rambaut A, Drummond AJ (2009) Tracer, version 1.5. http://beast.bio.ed.ac.uk/Tracer.

[66] Drummond AJ, Rambaut A (2007) BEAST: Bayesian evolutionary analysis by sampling trees. BMC Evolutionary Biology 7: 214 17996036 10.1186/1471-2148-7-214 PMC2247476

[67] Nei M (1987) Molecular evolutionary genetics. Columbia University Press, New York.

[68] Excoffier L, Laval G, Schneider S (2005) Arlequin ver 3.01. An integrated software package for population genetics data analysis. Evolutionary Bioinformatics Online 1: 47–60. PMC2658868 19325852

[69] Miller MP (2005) Alleles in space: Computer software for the joint analysis of interindividual spatial and genetic information. Journal of Heredity 96: 722–724. 16251514 10.1093/jhered/esi119

[70] Mantel N (1967) The detection of disease clustering and a generalized regression approach. Cancer Research 27: 209–220. 6018555

[71] Tajima F (1989) The effect of change in population size on DNA polymorphism. Genetics 123: 598–601. 10.1093/genetics/123.3.597 PMC1203832 2599369

[72] Fu XY (1997) Statistical tests of neutrality of mutations against population growth, hitchhiking and background selection. Genetics 147: 915–925. 9335623 10.1093/genetics/147.2.915 PMC1208208

[73] Ramos-Onsins SE, Rozas J (2002) Statistical properties of new neutrality tests against population growth. Molecular Biology and Evolution 19: 2092–2100. 12446801 10.1093/oxfordjournals.molbev.a004034

[74] Librado P, Rozas J (2009) DnaSP v5: A software for comprehensive analysis of DNA polymorphism data. Bioinformatics 25: 1451–1452. 10.1093/bioinformatics/btp187 19346325

[75] Wall J D, Hudson RR (2001) Coalescent simulations and statistical tests of neutrality. Molecular Biology and Evolution 18: 1134–1135. 11371601 10.1093/oxfordjournals.molbev.a003884

[76] Drummond AJ, Rambaut A, Shapiro B, Pybus OG (2005) Bayesian coalescent inference of past population dynamics from molecular sequences. Molecular Biology and Evolution 22: 1185–1192. 15703244 10.1093/molbev/msi103

[77] Heled J, Drummond AJ (2010) Bayesian inference of species trees from multilocus data. Molecular Biology and Evolution 27: 570–580. 10.1093/molbev/msp274 19906793 PMC2822290

[78] Wolfe KH, Li WH, Sharp PM (1987) Rates of nucleotide substitution vary greatly among plant mitochondrial, chloroplast, and nuclear DNAs. Proceedings of the National Academy of Sciences USA 84: 9054–9058. 10.1073/pnas.84.24.9054 PMC299690 3480529

[79] Huson DH, Bryant D (2006) Application of phylogenetic networks in evolutionary studies. Molecular Biology and Evolution 23: 254–267. 16221896 10.1093/molbev/msj030

[80] Pritchard JK, Stephens M, Donnelly PJ (2000) Inference of population structure using multilocus genotype data. Genetics 155: 945–959. 10835412 10.1093/genetics/155.2.945 PMC1461096

[81] Pritchard JK, Wen W (2004) Documentation for STRUCTURE 2.0. Available from: <http://pritch.bsd.uchicago.edu>.

[82] Evanno G, Regnaut S, Goudet J (2005) Detecting the number of clusters of individuals using the software STRUCTURE: a simulation study. Molecular Ecology 8: 2611–2620. 10.1111/j.1365-294X.2005.02553.x 15969739

[83] Miller MP (1997) Tools for population genetic analysis (TFPGA) 1.3: A Windows program for the analysis of allozyme and molecular population genetic data. Department of Biological Sciences, Northern Arizona University: AZ, USA.

[84] Hijmans RJ, Cameron SE, Parra JL, Jones G, Jarvis A (2005) Very high resolution interpolated climate surfaces for global land areas. International Journal of Climatology 25: 1965–1978.

[85] Collins WD, Blackmon M, Bitz C, Bonan G, Bretherton CS, et al (2004) The community climate system model: CCSM3. Journal of Climate 19: 2122–2143.

[86] Hasumi H, Emori S (2004) K-1 coupled GCM (MIROC) description. Tokyo: Center for Climate System Research, University of Tokyo.

[87] Peterson AT, Papes M, Eaton M (2007) Transferability and model evaluation in ecological niche modeling: A comparison of GARP and MaxEnt. Ecography 30: 550–560.

[88] Lobo JM, Jiménez-Valverde A, Real R (2008) AUC: A misleading measure of the performance of predictive distribution models. Global Ecology and Biogeography 17: 145–151.

[89] Placzek C, Quade J, Betancourt JL, Patchett PJ, Rech JA, Latorre C, Matmon A, Holmgren C, English NB (2009) Climate in the dry central Andes over geologic, millennial, and interannual timescales. Annals of the Missouri Botanical Garden 96: 386–397.

[90] Méndez MA, Soto ER, Correa C, Veloso A, Vergara E, Sallaberry M, Iturra P (2004) Morphological and genetic differentiation among Chilean populations of *Bufo spinulosus* (Anura: Bufonidae). Revista Chilena de Historia Natural 77:559–567.

[91] Templeton AR (1998) Nested clade analyses of phylogeographic data: testing hypotheses about gene flow and population history. Molecular Ecology 7: 381–397. 9627999 10.1046/j.1365-294x.1998.00308.x

[92] Widmer A, Lexer C (2001) Glacial refugia: Sanctuaries for allelic richness, but not for gene diversity. Trends in Ecology & Evolution 16: 267–269. 11369091 10.1016/s0169-5347(01)02163-2

[93] Soltis DE, Gitzendanner MA, Strenge DD, Soltis PS (1997) Chloroplast DNA intraspecific phylogeography of plants from the Pacific Northwest of North America. Plant Systematics and Evolution 206: 353–373.

[94] Swenson NG (2006). GIS-based niche models reveal unifying climatic mechanisms that maintain the location of avian hybrid zones in a North American suture zone. Journal of Evolutionary Biology 19: 717–725. 16674568 10.1111/j.1420-9101.2005.01066.x

[95] Carstens BC, Richards CL (2007) Integrating coalescent and ecological niche modeling in comparative phylogeography. Evolution 61: 1439–1454. 17542851 10.1111/j.1558-5646.2007.00117.x

[96] Antonelli A, Sanmartin I (2011) Why are there so many plant species in the Neotropics? Taxon 60: 403–414.

[97] Folguera A, Orts D, Spagnuolo ML (2011) A review of Late Cretaceous to Quaternary palaeogeography of the southern Andes. Biological Journal of the Linnean Society 103: 250–268.

[98] Bickford D, Lohman DJ, Sodhi NS (2007) Cryptic species as a window on diversity and conservation. Trends in Ecology & Evolution 22: 148–155. 17129636 10.1016/j.tree.2006.11.004

[99] Sersic AN, Cosacov A, Cocucci AA, Johnson LA, Pozner R, Avila LJ, Sites JW, Morando M (2011) Emerging phylogeographical patterns of plants and terrestrial vertebrates from Patagonia. Biological Journal of the Linnean Society 103: 475–494.

[100] Avila LJ, Morando M, Sites JW (2006) Congeneric phylogeography: hypothesizing species limits and evolutionary processes in Patagonian lizards of the *Liolaemus boulengeri* group (Squamata: Liolaemini). Biological Journal of the Linnean Society 89: 241–275.

