# Supplementary figures and images for: Munroa argentina, a Grass of the South American Transition Zone, Survived the Andean Uplift, Aridification and Glaciations of the Quaternary
Source: PLoS One. 2015 Jun 25;10(6):e0128559. doi: 10.1371/journal.pone.0128559 (PMC4484249; doi:10.1371/journal.pone.0128559)

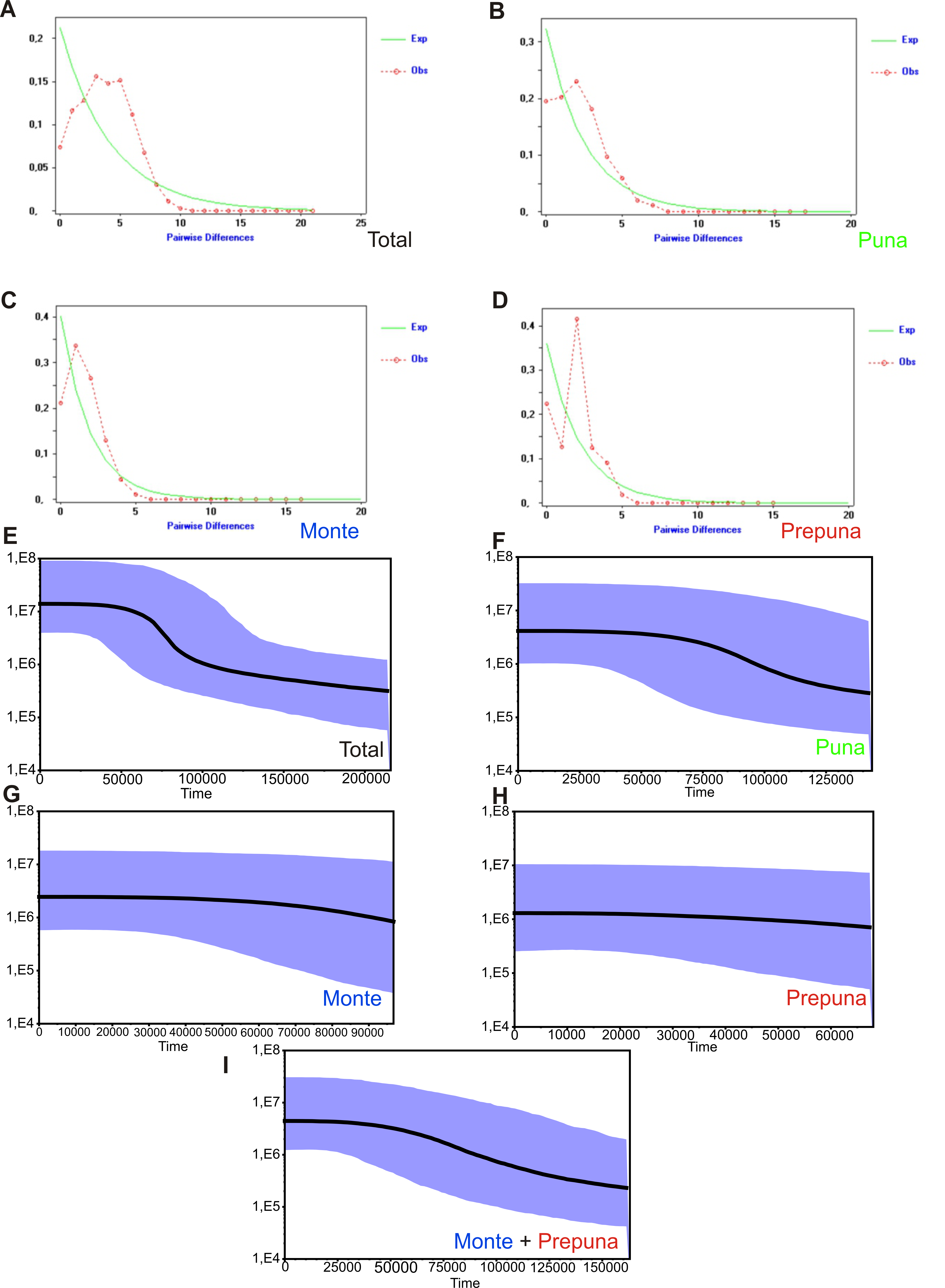

Supplement: S1 Fig — A-D, Mismatch distributions of pairwise nucleotide differences for population clusters of M. argentina. Dashed lines show the observed frequency distributions and solid lines show the distribution expected under the sudden-expansion model. E-I, Bayesian skyline semilog plots showing medians for the historical demographic trends for total populations of M. argentina. (TIF) [file pone.0128559.s001.tif]
