# Supplementary material for: Munroa argentina, a Grass of the South American Transition Zone, Survived the Andean Uplift, Aridification and Glaciations of the Quaternary
Source: PLoS One. 2015 Jun 25;10(6):e0128559. doi: 10.1371/journal.pone.0128559 (PMC4484249; doi:10.1371/journal.pone.0128559)
Supplement: S1 Table — (DOCX) [file pone.0128559.s002.docx]

**S1 Table.** Haplotype number (**H**) for *Munroa argentina*, outgroup taxa sampled, and GenBank accession numbers (the first *ndhA intron*, the second *rps16-trnK*).

| *Munroa argentina*: H13 KP091758, KP091799; H17 KP091759, KP091800; H15 KP091760, KP091801; H14 KP091761, KP091802; H8 KP091762, KP091803; H12 KP091763, KP091804; H5 KP091764, KP091805; H6 KP091765, KP091806; H7 KP091766, KP091807; H1 KP091767, KP091808; H2 KP091768, KP091809; H3 KP091769, KP091810; H4 KP091770, KP091811; H16 KP091771, KP091812; H10 KP091772, KP091813; H9 KP091773, KP091814; H11 KP091774, KP091815; H39 KP091775, KP091816; H36 KP091776, KP091817; H34 KP091777, KP091818; H41 KP091778, KP091819; H32 KP091779, KP091820; H30 KP091780, KP091821; H33 KP091781, KP091822; H31 KP091782, KP091823; H35 KP091783, KP091824; H38 KP091784, KP091825; H40 KP091785, KP091826; H37 KP091786, KP091827; H29 KP091787, KP091828; H18 KP091788, KP091829; H23 KP091789, KP091830; H20 KP091790, KP091831; H25 KP091791, KP091832; H26 KP091792, KP091833; H24 KP091793, KP091834; H22 KP091794, KP091835; H28 KP091795, KP091836; H27 KP091796, KP091837; H21 KP091797, KP091838; H19 KP091798, KP091839; *Munroa andina* GU359394,GU360722; *Munroa pulchella* GU359369, GU360505; *Swallenia alexandrae* GU359512, GU360639; *Scleropogon brevifolius* GU359530, GU360635; *Sohnsia filifolia* GU359531, GU360634. |
| --- |
