# Supplementary material for: Munroa argentina, a Grass of the South American Transition Zone, Survived the Andean Uplift, Aridification and Glaciations of the Quaternary
Source: PLoS One. 2015 Jun 25;10(6):e0128559. doi: 10.1371/journal.pone.0128559 (PMC4484249; doi:10.1371/journal.pone.0128559)
Supplement: S1 File — (DOCX) [file pone.0128559.s003.docx]

**S1 File**. Because an appropriate chloroplast nucleotide substitution rate for *M. argentina* has not been calibrated, we used the substitution rates 1.0 x 10^-9^ and 3.0 x 10^-9^ s/s/y (Wolfe et al. 1987) to estimate the effective population sizes (*Ne*) of each genetic group. Using DNASP with Jukes and Cantor correction, a substitution rate specific for the cpDNA loci in this study was also calculated as the sequence divergence divided by two lineages divided by two times the divergence time (3.4 Ma) among *M. argentina* populations to compare estimation of effective population sizes with those based on general rates from Wolfe et al. (1987). Wolfe KH, Li WH, Sharp PM (1987) Rates of nucleotide substitution vary greatly among plant mitochondrial, chloroplast, and nuclear DNAs. Proc Natl Acad Sci USA 84: 9054–9058.
